# COPD: the role of neutrophils in inflammation, pathophysiology, and as drug targets

**DOI:** 10.1042/CS20255452

**Published:** 2025-10-22

**Authors:** Michael Fricker, Ravi Lokwani

**Affiliations:** 1 School of Medicine and Public Health, University of Newcastle, Newcastle, New South Wales, Australia; 2 Asthma and Breathing Research Program, Hunter Medical Research Institute, Newcastle, New South Wales, Australia; 3 School of Pharmacy, Chapman University, Irvine, California, U.S.A.

**Keywords:** COPD, extracellular traps, inflammation, lung, neutrophil, protease, therapeutics

## Abstract

Neutrophils are innate immune effector cells that play a vital role in host defense against infection. They exert antimicrobial activities through degranulation, phagocytosis, intracellular killing, production of reactive oxygen species, and neutrophil extracellular traps. Dysregulation of neutrophil abundance, phenotype, and immune activity is a commonly observed inflammatory feature of chronic obstructive pulmonary disease (COPD), a chronic inflammatory disorder of the lungs with no effective cure. Here, we review the clinical association and involvement of neutrophils in COPD pathogenesis, progression, and exacerbation. The association between neutrophilia, airway microbial imbalance (dysbiosis), and clinical manifestations of COPD is described. We summarize the impact (or lack of) of current treatments, including inhaled corticosteroids, macrolide antibiotics, and phosphodiesterase inhibitors, on neutrophilic inflammation or neutrophilia-associated features. Finally, we review potential future therapeutic options to address neutrophilic inflammation in COPD currently in clinical development, including anti-alarmins and inhibitors of neutrophil mediators.

## Introduction

Neutrophils are innate immune cells that, along with eosinophils and basophils, are categorized as granulocytes, so-called due to their characteristic appearance, which features intracellular granules laden with immune effector molecules. They have been described as the first responders of the immune system. They are rapidly recruited to tissue sites of infection, injury, and in some cases allergy and are well known to play an essential role in the prevention of severe bacterial and fungal infections at barrier sites around the body, including the lung [1,2]. Neutrophils are the most abundant leukocyte in circulation, with an estimated 100 billion undergoing birth and maturation in the bone marrow on a daily basis [3]. Neutrophils are predominantly generated and mature in the bone marrow from myeloid progenitors. Maturation occurs via a series of intermediate states, during which neutrophils acquire their characteristic lobed nuclear morphology. Acquisition of various classes of preformed granules that contain key neutrophil effector molecules marks their transition through these states [4]. Azurophil granules indicate the transition from myeloblasts to promyelocytes and their contents include the neutrophil serine proteases neutrophil elastase (NE) and cathepsin G, myeloperoxidase (MPO), and antimicrobial defensins [5]. Specific granules occur in the myelocyte/metamyelocyte stage and contents include alkaline phosphatase, lysozyme, lactoferrin, and NADPH oxidase. Gelatinase granules are present in band cells, containing proteases, including cathepsins, matrix metalloproteinases (MMPs), and gelatinase. Secretory vesicles are detected in segmented cells, with membranes rich in proteins that become rapidly exposed at the cell surface following receipt of inflammatory stimuli that can mediate the establishment of firm contact with the vascular endothelium, such as the β2-integrin CD11b [5]. Mature post-mitotic neutrophils are retained or released from the bone marrow via a tug-of-war between CXC chemokine receptor 4 (CXCR4) signaling which promotes bone marrow retention, and CXCR2, which promotes egress into circulation. As neutrophils age in circulation, they up-regulate CXCR4 once more to promote reuptake into bone marrow for turnover [6]. Once in circulation, neutrophils must cross the endothelial barrier to reach sites of infection or injury. This is achieved in a highly directed manner through several stages, including initial rolling along the endothelial surface mediated by up-regulation of endothelial selectins in response to inflammatory signals. This is followed by firm adhesion mediated by integrins and intercellular adhesion molecule interactions in the lung and finally transendothelial migration into the tissue [7]. Neutrophil migration is directed through movement along a chemotactic gradient in response to an array of signals, which include chemokines such as CXC chemokine ligand 8 (CXCL8), bacterial products, lipid mediators, and complement factors [7]. These processes serve not only as a means to direct neutrophil migration to sites where they are needed but also to prime neutrophils for enhanced effector function once egress from circulation is complete. One such example of this is mechanical stress-induced priming of neutrophil bactericidal activity during migration to the trachea, mediated by activation of calcium signaling through the mechanosensitive ion channel Piezo1 [8].

### Neutrophil effector mechanisms

Once at the site of infection, neutrophils exert a range of innate immune effector mechanisms to contain and clear bacterial, fungal, and viral infections [1,2]. Neutrophils are highly competent phagocytes, and at sites of infection, will rapidly phagocytose opsonized and non-opsonized bacteria via Fc receptors, integrins, and other receptors. Once internalized, pathogens are rapidly neutralized by the concerted actions of enzymatic entities, including NADPH oxidase and MPO, that mediate reactive oxygen species (ROS) production, and granule-released antimicrobial peptides and proteases [2]. As these granule-based mediators are pre-synthesized, pathogen destruction occurs rapidly following internalization, while sparing the surrounding tissue from deleterious effects of granule contents. Neutrophil serine proteases and generation of ROS via oxidative burst are key microbicidal effector mechanisms [9]. Neutrophils may also undergo degranulation into the extracellular space, activating many of the same classical effector mechanisms against extracellular targets. Critically, this includes expulsion of mesh-like complexes of nuclear or mitochondrial DNA embedded with many neutrophil effector proteins called neutrophil extracellular traps (NETs). NETs have key antimicrobial and pro-inflammatory functions and are discussed further below. NET expulsion can be triggered by a number of mechanisms, often involving forms of necrotic death, including classical NETosis, pyroptosis, necroptosis, and even following secondary necrosis, which may occur in scenarios where the phagocytic turnover of apoptotic neutrophils is delayed [10–12]. NETs may also be expelled independently of cell death, and the resultant neutrophil cytoplasts, viable neutrophil ghosts devoid of nuclear DNA, are observed in increased numbers in bronchoalveolar lavage (BAL) of severe asthma patients. Neutrophil cytoplasts have been demonstrated to skew T cell responses toward a T helper 17 (Th17)-dominant phenotype, resulting in IL-17-dependent promotion of airway neutrophilia and poorer asthma outcomes [13]. Many of these NET expelling processes are dependent on the calcium-induced enzymatic activity of peptidyl arginine deiminase 4 (PAD4) [14].

## COPD: clinical associations of neutrophils

### COPD

Chronic obstructive pulmonary disease (COPD) is a common, chronic inflammatory airway disease that is a leading global cause of chronic morbidity and mortality. It is defined as a “heterogeneous lung condition characterized by chronic respiratory symptoms (including dyspnea, cough, expectoration, and/or exacerbations) due to abnormalities of the airways (bronchitis, bronchiolitis) and/or alveoli (emphysema) that cause persistent, often progressive airflow obstruction” [15]. While most commonly caused by exposure to cigarette smoke, other environmental factors such as biomass and air pollution exposure play an important role, particularly in low- and middle-income countries [16–18]. Various genetic risk factors have been identified for COPD, including mutations in SERPINA1, which result in a deficiency of alpha-1 antitrypsin (AAT), a key endogenous inhibitor of NE [19]. AAT deficiency is associated with the development of early-onset emphysema, directly implicating neutrophils and NE in the pathogenesis of this hallmark pathophysiological feature of COPD.

### Clinical associations of neutrophils in COPD

Airway and systemic inflammation are common features of COPD that promote pathogenesis, symptomatic burden, and susceptibility to exacerbations, including those associated with respiratory infection. Perhaps in keeping with the complex environmental, genetic, and temporal etiology of COPD and its varying clinical presentations, airway and systemic inflammation are heterogeneous, and this variation plays a key role in determining response to anti-inflammatory therapeutics, including inhaled corticosteroids (ICS; see section on therapeutics). Neutrophil dysregulation is the predominant inflammatory feature of COPD, with variations in neutrophil number, phenotype, and function observed in circulation and the large and small airway walls and lumens in the majority of COPD patients. COPD, like asthma, can be categorized by airway inflammatory phenotype, based on varying proportions of sputum eosinophils and neutrophils. This categorization includes eosinophilic (raised eosinophils), neutrophilic (raised neutrophils), mixed granulocytic (raised eosinophils and neutrophils), and paucigranulocytic (normal range proportions of eosinophils and neutrophils) phenotypes [20,21]. Eosinophilic COPD is a subset with elevated type-2 (T2) inflammation and favorable clinical response to ICS and the IL-4/-13 signaling blocker dupilumab [22–27]. Elevated sputum neutrophils, in stable disease, are associated with numerous aspects of the clinical burden of COPD. Neutrophilic COPD is the most common airway inflammatory phenotype, and increasing sputum neutrophils correlate with disease severity [28,29]. A comprehensive cluster analysis identified three COPD clusters, one of which was associated with intense systemic and airway neutrophilia and more severe lung function impairments [30]. Sputum neutrophilia in stable disease is associated with a predominance of bacterial exacerbations of COPD featuring increased pro-inflammatory mediators such as IL-1β and tumor necrosis factor-α (TNFα) [31]. In the eCOPD study, participants with above-median sputum neutrophil proportion had increased risk of severe exacerbation, in particular, in association with air trapping [32]. While blood eosinophils provide a reasonable surrogate marker of eosinophilic processes in the COPD airways, the blood neutrophil count is a poor indicator of sputum neutrophilia [33]. Despite this, the clinical significance of increased blood neutrophil count has been observed, with an association with more rapid lung function decline in COPD [34].

### Airway dysbiosis and neutrophilia in COPD

Multiple studies have reported clinically important associations between airway neutrophilia and disbalances in the airway microbial community, termed airway dysbiosis. In particular, overabundance of the proteobacteria *Haemophilus influenzae* has been linked to a distinct neutrophil-associated airway inflammatory pattern. Although airway dysbiosis is observed in association with neutrophilic inflammation, neutrophilia can occur independently of altered bacterial colonization in COPD [35].

A subset of COPD patients display rapid lung function decline, which is associated with altered lung microbiome. Mechanistically, airway dysbiosis is posited to promote rapid lung function decline through increased microbial production of the metabolite homocysteine, which in turn stimulates AKT kinase-mediated neutrophil activation. This reduces neutrophil apoptotic turnover, activates degranulation gene programs, and promotes pro-inflammatory NET formation by NETosis [36]. Airway dysbiosis featuring decreased bacterial diversity and a ‘biased’ profile is most prevalent in the neutrophilic inflammatory phenotype compared with eosinophilic, paucigranulocytic, and mixed granulocytic COPD, which predominantly display a ‘balanced’ microbial profile. Clustering revealed two major subsets of neutrophilic COPD with ‘biased’ or ‘balanced’ airway microbial profiles. The ‘biased’ profile is most commonly associated with dominance of the opportunistic pathogen *Haemophilus influenzae* and is associated with a specific profile of inflammatory mediators, including IL-1α, IL-1β, and TNFα. In contrast, neutrophilic COPD with a balanced microbial profile was characterized by elevated IL-17A, SAA (serum amyloid A), and IL-16 [28]. A study from Dicker and colleagues further described sub-phenotypes of COPD based on dominance of Proteobacteria (including the genus *Haemophilus*), Firmicutes, or neither (‘balanced’). COPD with a Proteobacteria-dominated microbiome had poorer lung function and experienced frequent exacerbations, while Firmicute dominance was associated with greater disease severity [37]. The proteobacteria, *Haemophilus*-dominated group was associated with increased expression of multiple neutrophil proteins, including MPO, catalase, MMP9, MMP8, and NE, again implicating neutrophilic inflammation in association with dysbiosis.

## Proposed roles of neutrophils in the pathogenesis of COPD

**Figure 1:**
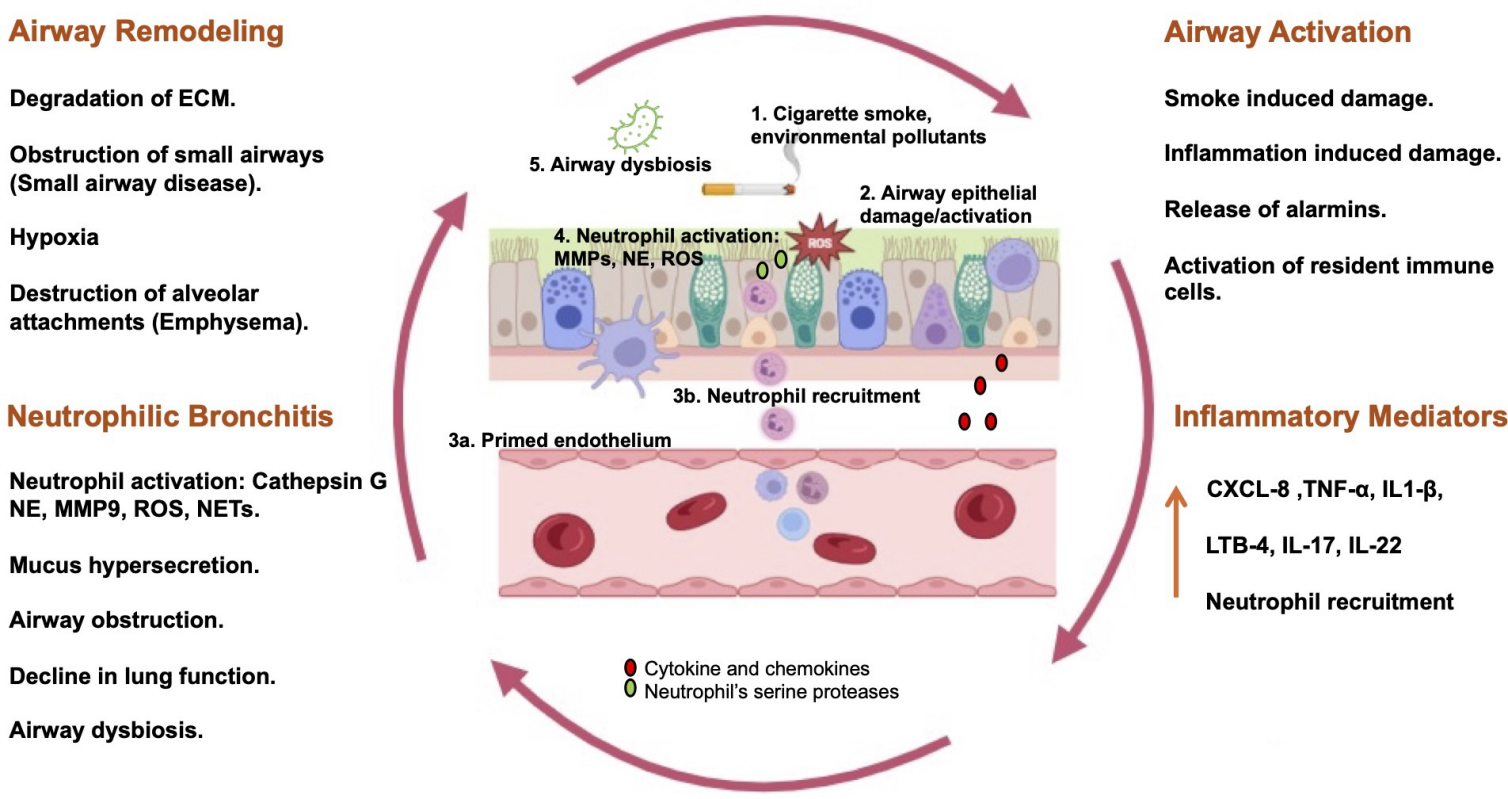
A self-perpetuating neutrophilic inflammatory cycle promotes pathophysiology of COPD. Noxious particles in cigarette smoke and air pollutants **(1)** cause activation and damage of airway epithelial/resident immune cells resulting in release of alarmins and chemotactic mediators **(2)** which then prime the endothelial wall **(3a)** and facilitate the recruitment of neutrophils into the airways **(3b)**. Excessive release of ROS and proteolytic enzymes, such as NE and MMP-9, by activated neutrophils in airways can lead to mucus hypersecretion, thus causing airway obstruction and decline in lung function **(4)**. Airway dysbiosis often co-occurs with a neutrophilic pattern of airway inflammation and relates to clinical outcomes including exacerbations and lung function decline **(5)**. Excessive neutrophil activation along with dysbiosis can further activate and/or damage the airway epithelial lining, which can cause airway remodeling and can precipitate the ‘vicious cycle’ of neutrophil-dominated airway inflammation in COPD, resulting in sustained neutrophil recruitment and airway damage even following smoking cessation. ECM: extracellular matrix. [Created in BioRender. Lokwani, R. (2025). https://BioRender.com/o78v738]**.**

As discussed above, airway neutrophilia is a common hallmark of COPD, and genetic deficiency of the endogenous AAT results in early development of emphysema [38]. Thus, neutrophils have been proposed to play a central role in the pathogenesis of COPD. The accumulation of neutrophils in the lungs has been linked with alveolar damage, reduction in lung function, and decline in gas exchange, as well as failure in clearance of potentially pathogenic bacteria from the airways [39]. Neutrophils can inflict pathological effects through production of a variety of cytotoxic products, including a potent array of serine proteinases such as NE, MMP9, MMP8, cathepsin G, and proteinase-3 [40]. The recruitment of neutrophils is driven by lung resident cells, which include epithelial cells, macrophages, innate lymphoid cells, and T cells. The pathogenesis of COPD is associated with the inhalation of noxious particles and gases, primarily cigarette smoke and smoke from wood fires used for cooking [41,42]. Cigarette smoke, containing harmful ROS such as carbon monoxide, activates damage-associated molecular pattern (DAMP) and pathogen-associated molecular pattern (PAMP) receptors such as toll-like receptors (TLRs) and NOD-like receptors present on the surface of epithelial cells, resident macrophages, dendritic cells, and resident lymphocytes [CD8^+^, CD4^+^ (Th1 and Th17)] [43]. The activation of these receptors triggers the release of chemoattractants such as TNFα, IL-1β, IL-6, granulocyte-monocyte colony-stimulating factor (GM-CSF), C-C motif chemokine ligand 2 (CCL2), ROS, leukotriene B4 (LTB4), and CXCL8 by these cells, which then initiate the recruitment of circulating neutrophils [40]. Cigarette smoke can also induce the release of IL-33 (a potent DAMP cytokine and part of the alarmin cytokine family) by damaged epithelial cells, which can then activate resident innate lymphoid cells (ILCs), mainly type 1 and 3 (ILC1 and ILC3). The activated ILCs can further contribute to secretion of chemoattractants that can recruit neutrophils from circulation [44,45]. Neutrophils then follow this chemoattractant gradient to the site of injury. The repeated inhalation of cigarette smoke or airway pollutants can result in excessive infiltration of neutrophils in airways. The chemotactic milieu present at the site of inflammation not only promotes recruitment and activation of neutrophils but also prolongs the lifespan of neutrophils [46]. In the airway, activated neutrophils undergo degranulation involving the release of granular contents, including serine proteases such as NE, MMP9, and cathepsin G. While these enzymes play a key role in neutrophil antimicrobial activity after containment of pathogen in the neutrophil phagosome, excessive activation can unleash them in the extracellular compartment, which can be damaging for host cells. In the case of COPD, release of NE by activated neutrophils in airways results in degradation of extracellular matrix components such as elastin, fibronectin, and collagen [38]. Recent studies suggest that in COPD, neutrophils can overcome the pulmonary endogenous antiprotease barrier by releasing AAT-resistant NE via exosomes [47]. The damage of airway stromal cells by these proteases results in further release of neutrophil chemoattractants such as CXCL8, facilitating further neutrophil recruitment in airways in a posited cycle of neutrophil-induced tissue damage and tissue damage-induced neutrophil recruitment. The dumping of ROS and NE in the airways by neutrophils can promote mucus hypersecretion. This occurs by activation of TACE (TNFα converting enzyme)-mediated TGFα (transforming growth factor) release, which in turn activates epithelial growth factor receptor (EGFR) through mitogen-activated protein kinases that subsequently increase expression of mucin genes MUC5AC and MUC5B, resulting in increased mucus production and goblet cell submucosal gland hyperplasia [40,48]. The continuous recruitment of neutrophils in the airway due to the establishment of a self-sustaining vicious cycle (as shown in Figure 1) and mucus hypersecretion establishes chronic bronchitis in airways, one of the hallmarks of COPD that causes airway obstruction leading to a decline in lung function [49]. The potential cyclic and self-perpetuating nature of this bronchitis can persist even beyond cessation of smoking. The destruction of airways by neutrophil proteases and ROS is known to cause airway remodeling in COPD, which can take the form of two major pathologies, i.e. small airway disease and emphysema, which can be confirmed with computed tomographic scans [50]. Small airway disease precedes emphysema [40] and features blockages of small airways (<2 mm in diameter).

Progression of inflammation can further result in destruction of the alveolar attachments on the outer wall of small airways [51]. While neutrophils can be present in higher numbers in aspirates of conducting airways, the aspirates of small airways generally have a greater number of macrophages [52] that are mainly activated by the dendritic cells in response to cigarette smoke and other stimuli. Alveolar macrophages release inflammatory mediators such as IL-6, CXCL8, MCP1 (monocyte chemotactic peptide), LTB4, ROS, and proteolytic enzymes (MMP9 and MMP12) [53]. These mediators act as chemoattractants for the entry of neutrophils that further increase the oxidative burden of the airways by releasing potent ROS such as H_2_O_2_ and proteolytic enzymes such as NE, cathepsin G, and proteinase 3 (PR3) [54]. Alveolar macrophage release of neutrophil chemoattractants such as CXCL8 is corticosteroid insensitive in COPD, potentially contributing to the lack of impact of ICS treatment in neutrophilic COPD [55]. Small airway disease can even be present in mild COPD patients [56].

The progression of small airway disease can take a more severe form in COPD known as emphysema, which is defined as the expansion of distal airspaces beyond the terminal bronchioles due to the destruction of airway walls [57]. COPD patients with emphysema generally show neutrophil-based inflammation with elevated levels of neutrophil chemoattractants such as CXCL8, MPO, and LTB4, which promotes persistence of neutrophils in the airways of these patients [58]. The proteases and ROS released by neutrophils contribute to pathogenesis by causing destruction of alveolar cells and lung parenchyma, precipitating emphysema [59]. Emphysematous lung destruction contributes to decreases in expiratory airflow by decreasing the elastic recoil force that drives air out of the lungs.

## Altered neutrophil phenotype and function in COPD

Many aspects of neutrophil biology and effector functions have been described to be dysregulated in COPD. Around 50% of COPD patients feature neutrophil-dominated chronic bronchitis [60] that negatively correlates with patients’ lung function [39,46]. A recent study by Kapellos et al. characterized COPD neutrophils by single-cell sequencing and reported that blood neutrophils in COPD patients featured an altered phenotype with decreased interferon gene expression, critical for viral clearance, and up-regulated expression for genes associated with alarmins, degranulation, Rho GTPase signaling, and ephrin signaling, which can promote airway inflammation and mucus hypersecretion. Along with this, resistin, a gene known to inhibit bacterial clearance, was also up-regulated in COPD patients [39]. Chemotactic behavior of neutrophils is intrinsically altered in COPD, with greater migratory speed but reduced chemotactic accuracy that appears to be driven by a phosphoinositide 3-kinase (PI3K) -dependent signaling process [61,62]. Our prior study demonstrated the presence of an activated neutrophil phenotype featuring low CD62L/L-selectin surface expression in the circulation of COPD patients. Interestingly, we did not observe this phenotype in asthma patients, suggesting that the presence of activated neutrophils in COPD could be a result of systemic inflammation, more common in COPD patients. COPD patients have also been reported with the presence of an altered neutrophil phenotype in their airways featuring dysfunctional phagocytosis leading to airway bacterial colonization [63]. A recent study reported an increased presence of a morphologically distinct neutrophil phenotype featuring a hypersegmented nucleus in the airways of obstructive airway disease patients, including COPD. The increased presence of these hypersegmented neutrophils was correlated with reduced lung function and airway obstruction in patients with obstructive airway disease [46]. Hypersegmented neutrophil subsets are reported to produce increased ROS production and to suppress T cell responses and apoptosis of gastric epithelial cells in *in vitro* studies [64,65]. A recent study from Lodge and colleagues demonstrated an altered degranulation of COPD neutrophils under hypoxic conditions, with systemic and tissue hypoxia a known feature of COPD. Degranulation under hypoxic conditions was associated with greater release of granule proteins, including NE and MPO, compared with normoxic conditions [66,67]. Of note, supernatants from COPD neutrophils that underwent degranulation under hypoxic conditions showed enhanced ability to promote neutrophil rolling along endothelial monolayers, consistent with a self-perpetuating cycle of tissue damage-induced enhancement of neutrophil recruitment and activation in the airways, in turn promoting further damage [67]. Increased release of NETs by airway neutrophils has been demonstrated by several groups, correlating with disease severity [68–72]. These alterations in neutrophil phenotype can be a disease-associated feature of COPD, as exposure to high concentrations of inflammatory cytokines in the airways can not only prolong neutrophil survival but can also change their phenotype, as previously shown in the case of acute respiratory distress syndrome (ARDS) [73]. Increased airway NETs have been associated with a type 1 inflammatory signaling milieu, including IL-6 trans-signaling, as well as increased dominance of *Haemophilus* bacteria and reduced airway microbial diversity [69,72]. In addition, increased sputum NETs were associated with reduced neutrophil phagocytosis of bacteria, suggestive of an altered pro-inflammatory and microbial signaling environment that triggers prolonged and inappropriate damaging neutrophil effector mechanisms such as NET release and release of granule contents.

## Role of neutrophils in exacerbations of COPD

Acute exacerbation of COPD (AECOPD), involving periodic worsening of symptoms, is associated with reduced quality of life [74], accelerated lung function decline [75], and increased mortality [76]. Those COPD patients who experience frequent AECOPD have the poorest prognosis [77,78]. Prevention of episodes of AECOPD is thus considered a key target for development of therapeutic interventions that can improve long-term prognosis and halt progression of the disease. AECOPD is often associated with the presence of viral or bacterial infection [31]. Both viral and bacterial triggers of AECOPD are associated with increases in airway and circulating neutrophilia [31,79]. The neutrophilic COPD subtype featuring increased airway *Haemophilus influenzae* (see above) is notably stable during AECOPD episodes, while neutrophilic COPD with a ‘balanced’ airway microbial profile was associated with greater variation in inflammatory and microbial patterns during exacerbation [28]. Increased blood neutrophil counts in stable COPD are associated with increased risk of severe exacerbations, and when measured during AECOPD, can predict adverse outcomes [80,81]. AECOPD is associated with altered circulating neutrophil phenotype, consistent with priming and increased recruitment and homing of neutrophils from circulation to the airway tissue [82,83]. In models of experimental rhinovirus infection, increased sputum and bronchial neutrophils are observed in COPD patients, but not in non-COPD controls, demonstrating a predisposition to excessive airway neutrophil recruitment following airway viral infection in COPD [82,84]. Increased presence of DAMPs in circulation during AECOPD is accompanied by increased neutrophil expression of DAMP/PAMP receptors (e.g. Toll-like receptors TLR2 and TLR4) and reduced serum levels of the decoy receptor for advanced glycation endproduct (sRAGE), suggesting responses to tissue damage cues from infected COPD lungs can promote the exaggerated neutrophil responses observed in the airways during exacerbations [85]. Activation of neutrophil effector mechanisms in the airways is increased in AECOPD vs. stable COPD and when compared with healthy controls experiencing lung infections. This includes increased presence of degranulation products such as NE and increased production of ROS by airway and systemic neutrophils, which are particularly associated with, but not restricted to, airway bacterial infection in COPD [82,86,87]. A recent study from Katsoulis and colleagues demonstrated increased airway NETs in COPD vs. healthy smokers or non-smokers following experimental rhinovirus infection, along with demonstration of increased sputum NETs during AECOPD in a longitudinal COPD cohort [88]. In preclinical mouse models of AECOPD, the authors observed increased airway NETs without evidence of tissue damage or necrotic cell death, suggesting activation of specific NET-releasing pathways. In support of this, systemic administration of a PAD4 inhibitor reduced NETs and extracellular dsDNA in this model and inhibited accumulation of BAL inflammatory cells, chemokines, and cytokines. This suggests that excessive NET release during AECOPD may play an important role in promoting exaggerated and deleterious airway inflammatory responses to infection in COPD.

## Therapeutic options targeting neutrophilic inflammation in COPD

### Current options

#### Inhaled corticosteroids and bronchodilators

Analysis of large clinical trial datasets indicates clinical benefit in terms of reduction in exacerbations by ICS. Treatment benefit is limited to patients with elevated blood eosinophil counts [89,90]. This is reflected in clinical guidelines, which recommend ICS use in COPD only in those who experience exacerbations despite ongoing long-acting beta-2 agonist-long-acting muscarinic antagonist (LABA-LAMA) therapy and who have an elevated blood eosinophil count [15]. Neutrophilic inflammation is corticosteroid insensitive in COPD, including a corticosteroid-insensitive alveolar macrophage – CXCL8 – neutrophil axis [55]. Corticosteroids may, in fact, promote neutrophilia through actions including suppression of neutrophil apoptosis and clearance [91]. A recent study suggested that macrophage migration inhibitory factor (MIF) promotes glucocorticoid resistance of airway neutrophilic inflammation through cleavage of annexin-A1. Annexin-A1 is a glucocorticoid-induced factor that can suppress neutrophil accumulation in the lungs, and thus MIF antagonism may represent a means to enhance glucocorticoid sensitivity of neutrophilic inflammation in COPD [92,93]. Thus, ICS are not currently recommended in non-eosinophilic COPD and are likely ineffective to prevent neutrophil-associated pathophysiology. Addition of bronchodilatory LAMA to ICS-LABA in triple therapy provides superior clinical benefit in COPD, including reduced exacerbations [94]. *In vitro* and preclinical animal model studies have suggested LAMA, in particular tiotropium, can exert anti-inflammatory effects [95]. Numerous immune cells, including neutrophils, express key muscarinic receptors M1–M3. Studies of the impact of LAMA on neutrophil inflammatory functions in the context of COPD and asthma have produced mixed results, and currently, evidence to support a clinical impact of LAMA beyond bronchodilation is lacking [95].

#### Macrolides

Current GOLD guidelines advocate for the use of low-dose macrolide azithromycin as an add-on therapy in ex-smokers with below-threshold blood eosinophils who experience exacerbations despite treatment with LABA-LAMA or triple inhaled ICS-LABA-LAMA therapy [15,94]. Azithromycin is a macrolide antibiotic with demonstrated efficacy in reducing exacerbations in both COPD and severe asthma [96–98]. Macrolides can exert numerous potentially relevant biological effects that could reduce exacerbation frequency in chronic inflammatory respiratory diseases, including antibacterial, anti-inflammatory, and antiviral effects. A smaller placebo-controlled trial in COPD participants with persistent neutrophilic bronchitis demonstrated a clinically significant reduction in severe exacerbations, with non-significant trends toward decreases in sputum neutrophil proportion, CXCL8 concentration, and bacterial load [99]. Larger randomized controlled trials (RCTs) have not addressed the impact of macrolides on airway inflammation in COPD. Azithromycin treatment of neutrophilic COPD results in broad alterations of gene expression programs both in sputum and circulation, consistent with a suppressive effect on interferon and T-cell responses [100]. Studies in asthma have also demonstrated reduction in *H. influenzae* load and reduction in innate immune mediators, including TNFα, IL1β, and IL6, in particular in neutrophilic or non-eosinophilic phenotypes [101–103]. Thus, azithromycin may prevent exacerbations in COPD by broad immunomodulatory activities, including suppression of neutrophil-related microbial and inflammatory factors, although significant reductions in airway neutrophil numbers may not be observed (Table 1).

**Table 1:** Current therapeutic options that may affect neutrophilic inflammation in COPD.

Drug	Drug target	Target population	Guideline endorsed?	Route
Macrolides	Airway dysbiosis including *H. influenzae* Innate inflammation related to neutrophilia Potential anti-viral impacts	Ex-smokers who experience exacerbations despite LABA-LAMA or ICS-LABA-LAMA	GOLD	Oral
Roflumilast	PDE4 Inhibits neutrophil ROS production Inhibits TNF production by macrophages/monocytes	FEV1<50% & chronic bronchitis, with ongoing exacerbations despite ICS-LABA-LAMA or LABA-LAMA	GOLD	Oral
Ensifentrine	PDE3/4 PDE3 activity promotes bronchodilatory effect PDE4 may exert anti-inflammatory effect. Reduced sputum neutrophil influx following LPS challenge in healthy volunteers	Ongoing dyspnea despite LABA and/or LAMA treatment	GOLD	Inhaled-nebulized

FEV1 = forced expiratory volume in 1 second. GOLD = Global Initiative for Chronic Obstructive Lung Disease. ICS = inhaled corticosteroid. LABA = long-acting beta-2 agonist. LAMA = long-acting muscarinic antagonist. LPS = lipopolysaccharide. PDE, phosphodiesterase.

#### Phosphodiesterase (PDE) inhibitors

Phosphodiesterases (PDE) are enzymes involved in the turnover of the signaling molecule cAMP. PDE3/4 are widely expressed in the lung airway smooth muscle and PDE4 also in airway epithelium and immune cells. They are considered important drug targets for inhibition, as increasing cAMP in airway smooth muscle can produce a bronchodilatory effect and in immune cells can inhibit inflammatory signaling (Table 1). Roflumilast is an oral PDE4 inhibitor with anti-inflammatory properties that reduces exacerbations in patients with a history of exacerbations and chronic bronchitis. PDE4 inhibitors can exert a range of anti-inflammatory effects relevant to the neutrophilic inflammatory milieu in COPD, including inhibition of TNF production by alveolar macrophages and monocytes and inhibition of ROS production by neutrophils [104]. However, side effects, particularly affecting the GI tract, often result in cessation of treatment. In an effort to address this, inhaled PDE inhibitors have been developed, aiming to increase localized lung exposure while minimizing systemic-exposure-related side effects. Ensifentrine is an inhaled dual PDE3/4 inhibitor recently approved for use in the U.S.A. [104]. It is included in the GOLD guidelines as a recommended treatment for patients who experience ongoing dyspnea despite treatment with LABA and/or LAMA [94]. Of note, ensifentrine reduces exacerbations in COPD trial populations, and whether this relates to either PDE3 and/or PDE4 inhibitory activities remains unclear, as does the therapeutic potential for ensifentrine in exacerbation-prone COPD patients [105]. A phase I study in healthy volunteers demonstrated ensifentrine reduced sputum inflammatory cell counts, including neutrophils, compared with placebo in a lipopolysaccharide challenge model; thus, impacts on neutrophil-related inflammation are possible in certain contexts, particularly relating to bacterial infection [106]. Further studies to elucidate the relative impact of inhaled PDE3/4 inhibition in the context of stable and acute neutrophilic inflammation in COPD are required.

### Potential future treatments

- *Inhaled PDE4 inhibition:* CHF6001 (Tanimilast) is an inhaled PDE4 inhibitor currently in a phase 3 clinical trial for COPD patients with a history of exacerbations despite ongoing triple inhaled therapy (Table 2). CHF6001 treatment decreased sputum inflammatory markers in COPD, including neutrophil chemoattractants CXCL8, LTB4, MMP9, and TNFα; however, no impact on sputum neutrophil count or markers of neutrophil activity (NE and MPO) was observed [107]. The anti-inflammatory impacts, including reduced sputum macrophage numbers, are consistent with a suppression of macrophage-related inflammatory processes by this treatment rather than direct impacts on neutrophils. Evidence supports greater effects of PDE4 inhibitors in eosinophilic COPD, suggesting this may not be the optimal strategy for non-T2 neutrophilic subtypes of COPD [104].
- *Anti-alarmins:* alarmins are a group of cytokines that can be released from epithelial cells following stimulation by various environmental stressors, including pollutants, viruses, bacteria, and allergens that can promote both T2 and non-T2 inflammation [108]. Tezepelumab is a biologic that inhibits thymic stromal lymphopoietin (TSLP), currently approved for severe asthma. TSLP can stimulate both Th2- and Th1-related inflammation; thus, TSLP blockade has potential broad clinical use across COPD inflammatory phenotypes. In the recently published COURSE phase 2a trial, Tezepelumab did not meet the primary outcome of reducing exacerbations in a population of COPD treated with triple inhaled maintenance therapy with a history of frequent exacerbations [109]. Pre-specified analyses by blood eosinophil count revealed a trend toward greater exacerbation reduction in patients with increasing blood eosinophil and FeNO (fractional exhaled nitric oxide) T2 biomarker measures, in particular blood eosinophils above 150/μL, which has informed ongoing phase 3 trial design (Table 2). In contrast, no impact was seen in patients with blood eosinophils below 150/μL, with a numerically higher rate of exacerbations observed compared with placebo in this group. These results differ slightly from severe asthma, in which tezepelumab was the first biologic to display broad therapeutic benefit irrespective of baseline T2 biomarker status, albeit with greater exacerbation reduction observed in T2 high patients [110]. Further trials will be required to understand any potential clinical benefit for TSLP blockade in T2 low COPD populations, and studies incorporating airway biospecimen analysis are required to understand the impact on neutrophil-related inflammation and phenotypes. In severe asthma bronchoscopy studies, tezepelumab therapy had no impact on sputum or airway wall neutrophil counts [111,112]. An additional TSLP-blocking biologic therapy, solrikitug, is currently in the early stage of phase 2 for COPD with elevated blood eosinophil counts (Table 2).
  The alarmin IL-33 can be released during tissue damage triggered by environmental factors, including pollutants and pathogens. In the reduced form, it signals via the interleukin-1 receptor-like 1 (IL1RL1/ST2) receptor complex, while oxidized IL-33 preferentially binds the sRAGE/EGFR complex. Expression of IL-33 is increased in COPD and associated with disease severity [113]. Stimulation of human endothelial cells with exogenous IL-33 results in activation of a type 1 inflammatory response and production of a cytokine and chemokine profile consistent with increased recruitment of neutrophils [114]. Evidence regarding neutrophil expression of IL-33 receptors and responsiveness to IL-33 is mixed. A recent study revealed increased expression of IL1RL1/ST2 on neutrophils in a preclinical model of asthma following rhinovirus infection and increased production of NETs and type 1 and 2 cytokines by isolated blood neutrophils in these conditions following IL-33 stimulation [115]. Thus, IL-33 may potentially promote neutrophilia and, in certain contexts such as infection and inflammatory airway disease, promote neutrophil activation and NET release. Currently, several biologics targeting the alarmin IL-33 (itepekimab and tozarakimab) or the receptor ST2 (astegolimab) are undergoing phase 2/3 trials in COPD. In a phase 2 trial, the IL-33 blocker itepekimab reduced exacerbations and improved lung function in a subgroup of COPD patients who were former smokers, irrespective of baseline blood eosinophil status [116]. Tozorakimab exhibits a dual mechanism of action, binding both reduced and oxidized forms of IL-33 to inhibit signaling via ST2 and RAGE/EGFR complex pathways [117]. Astegolimab did not significantly reduce exacerbation frequency in a phase 2 COPD trial. Post-hoc sub-analyses indicated favorable, albeit non-significant, exacerbation rate reductions with astegolimab associated with lower blood and sputum eosinophil counts. Recently announced topline results from itepekimab and astegolimab phase 3 trials indicate failure to meet the primary outcome in 2 out of 4 trials – further analyses and trials to identify responsive sub-groups would appear to be required. These RCTs did not use inflammatory biomarkers for participant selection (Table 2), thus may have included T2 low and neutrophilic COPD patients. Further RCTs will be required to establish clinical benefit in these T2 low and potentially neutrophilic phenotypes of COPD [118]. As is the case for TSLP inhibition, clinical studies assessing airway biospecimens and neutrophil-related outcomes are needed to determine the impact of IL-33 blockade on neutrophil-related processes and clinical outcomes in COPD (Table 2).
- *Targeting neutrophil chemotaxis and mediators:* to date, treatments that have directly targeted neutrophil chemotaxis and type 1 inflammation via TNFα have not been successful in COPD trials [119,120]. These include CXCR2 antagonism, which can reduce airway neutrophilia, and had mixed results on lung function and resulted in discontinuation in some patients due to low absolute neutrophil count [121–123]. The cytokine IL-17A induces neutrophilia-promoting factors such as IL-6, IL-8, GM-CSF, and granulocyte colony stimulating factor (GCSF) and is increased in serum, bronchial biopsies, and sputum in COPD. CNTO 6785, a monoclonal antibody therapy targeted to IL-17A, failed to improve lung function or symptom burden in a placebo-controlled trial in COPD [124].
  A phase 2 trial of an oral NE inhibitor (AZD9668) did not improve lung function, symptomatic burden, or serum biomarkers of type 1 inflammation [125], potentially due to a lack of target engagement in the lungs, with no significant impact on sputum neutrophil counts or NE activity reported in a separate bronchiectasis trial [126]. Inhaled inhibitors of NE (CHF6333 and POL6014) are in development and currently in early-stage trials for cystic fibrosis and bronchiectasis (NCT04010799, NCT06166056, and NCT03748199), with rapid inhibition of sputum NE activity evident in CF patients after a single dose at multiple concentrations [127]. An alternative means to inhibiting neutrophil serine proteases, including NE, is via inhibition of dipeptidyl peptidase 1 (DPP-1), which mediates activation of neutrophil serine proteases. DPP-1 inhibitor brensocatib in bronchiectasis significantly delays time to first exacerbation and results in an overall reduction in exacerbation rate, with confirmation of reduced sputum NE activity [128]. Broad inhibition of sputum neutrophil serine proteases, including NE, PR3, and cathepsin G by brensocatib treatment was confirmed in follow-up analyses [129]. Brensocatib recently received FDA approval for bronchiectasis. This therapeutic approach is yet to be tested in COPD; however, the broad inhibitory activity of brensocatib on several neutrophil proteases known to be dysregulated in COPD holds potential. An additional oral DPP-1 inhibitor, HSK31858, shows promise for bronchiectasis after a positive phase 2 trial. A current phase 2 study is testing its efficacy in patients with asthma, bronchiectasis, or COPD with evidence of mucus hypersecretion (Table 2).
  An oral inhibitor of MPO, mitiperstat, is currently in phase 2 trials for moderate-to-severe COPD, with the primary outcome a measure of composite exacerbation events (NCT05492877). Mitiperstat displays a greater potency for extracellular vs. intracellular MPO; thus, it is hoped it will inhibit inflammatory extracellular ROS-inducing activities of MPO while preserving its intragranular microbicidal function [130].
  Excessive NET release in the airways can enhance mucus viscosity and stimulate innate immune responses through increased presence of extracellular DNA (eDNA). A recent pilot unblinded trial of nebulized recombinant human deoxyribonuclease I/dornase alfa in asthma and COPD patients pre-screened for high levels of sputum eDNA demonstrated a significant decrease in eDNA levels compared with placebo, and an improvement of COPD symptom score, thus may merit further investigation in larger populations [131].

**Table 2:** Potential future treatments for COPD that could affect neutrophil-related inflammation.

Potential treatment	Drug target	Trial population	Biomarker participant selection	Route	Impacts neutrophils?	Trial reference
CHF6001/Tanimilast	PDE4	FEV1<50% and FEV1/FVC<0.7 with history of moderate/severe exacerbation despite ICS-LABA-LAMA.	No biomarker	Inhaled	↓CXCL8, LTB4, MMP9, TNFα. [102] NE, MPO, sputum neutrophil count unchanged. Likely affecting macrophages.	Phase 3; NCT04636814
Tezepelumab	TSLP	Mod-very severe COPD with history of moderate/severe exacerbation despite triple or dual inhaled COPD therapy	Blood eosinophils ≥150 /μL	Injected	Unknown (lack of clinical evidence)	Phase 3; NCT06883305, NCT06878261
Solrikitug	TSLP	Symptomatic COPD despite triple or dual inhaled COPD therapy	Elevated blood eosinophils	Injected	Unknown (lack of clinical evidence)	Phase 2; NCT06496620
Itepekimab	IL-33	Former smokers with mod-severe COPD with chronic bronchitis and history of moderate/severe exacerbation despite triple or dual inhaled COPD therapy	No biomarker	Injected	Unknown (lack of clinical evidence)	Phase 3; NCT04701983, NCT04751487
Tozorakimab	IL-33 (reduced and oxidized forms signaling via ST2 and RAGE/EGFR respectively)	Symptomatic COPD with history of moderate/severe exacerbation despite triple or dual inhaled COPD therapy	No biomarker	Injected	Unknown (lack of clinical evidence)	Phase 3; NCT05166889, NCT05158387, NCT06040086
Astegolimab	ST2 (IL-33 receptor)	Symptomatic COPD with history of moderate/severe exacerbation despite triple or dual inhaled COPD therapy	No biomarker	Injected	Unknown (lack of clinical evidence)	Phase 3; NCT05037929, NCT05595642
Brensocatib	DPP-1	Reduced exacerbations in phase 2 and phase 3 bronchiectasis trial populations	No biomarker	Oral	Reduced sputum NE and neutrophil serine protease activity in bronchiectasis	No current COPD RCTs
HSK31858	DPP-1	Patients with asthma, bronchiectasis, or COPD with mucus hypersecretion	Elevated sputum production	Oral		Phase 2; NCT06820749
Mitiperstat	MPO	COPD with history of moderate/severe exacerbation despite triple or dual inhaled COPD therapy	No biomarker	Oral	Inhibits blood MPO activity	Phase 2; NCT05492877

CXCL = chemokine (C-X-C motif) ligand. DPP-1 = dipeptidyl peptidase-1. FEV1 = forced expiratory volume in 1 second. FVC = forced vital capacity. ICS = inhaled corticosteroid. LABA = long-acting beta-2 agonist. LAMA = long-acting muscarinic antagonist. LTB4 = leukotriene B4. MMP = matrix metalloprotease. MPO = myeloperoxidase. NE = neutrophil elastase. PDE = phosphodiesterase. RAGE = receptor for advanced glycation endproducts. TNF = tumour necrosis factor. TSLP, thymic stromal lymphopoietin.

## Conclusions

Extensive dysfunction of neutrophils has been described in COPD, affecting almost every facet of the neutrophil immune responses from chemotaxis and accumulation in the lung, through to aberrant and chronic activation of neutrophil effector mechanisms such as degranulation, ROS production, and NET release. An increasing weight of evidence suggests an important mechanistic contribution of this chronic pulmonary and systemic neutrophil activation to the pathogenesis and pathophysiology of COPD and AECOPD. An emerging area of neutrophil research relates to their potential roles in mediating tissue repair, including in the lung; however, this remains unexplored in COPD [2]. Current COPD treatments, macrolides and PDE inhibitors may exert anti-inflammatory (and for macrolides antimicrobial) effects that lessen the amplitude of neutrophil-related inflammatory processes in the COPD airways. The mechanistic importance of this, however, remains to be elucidated. Several anti-alarmin treatments are currently in advanced trials for COPD, with their potential to modulate neutrophil-related inflammation currently unclear. Strategies that directly reduce neutrophil chemotaxis, such as CXCR2 antagonism, have not advanced due to a lack of clear clinical benefit and safety issues regarding neutrophil depletion. New approaches that broadly (e.g. brensocatib) or specifically target neutrophil immune effector dysregulation (e.g. inhaled NE inhibitors, MPO inhibitors, inhaled DNases) are promising therapeutic approaches currently in development. So are neutrophils key players and legitimate therapeutic targets in COPD, or merely cellular witnesses to other dominant pathogenic processes? The jury is still out, and the conclusion will rest to a certain extent in the success (or failure) of these novel therapeutic approaches currently in development.

## Open Access

This article has been published open access under our Subscribe to Open programme, made possible through the support of our subscribing institutions, learn more here: https://portlandpress.com/pages/open_access_options_and_prices#conditional

## Abbreviations

AAT: alpha-1 antitrypsin
AECOPD: acute exacerbation of COPD
BAL: bronchoalveolar lavage
COPD: chronic obstructive pulmonary disease
CXCL8: CXC chemokine ligand 8
CXCR4: CXC chemokine receptor 4
DAMP: damage-associated molecular pattern
DPP-1: dipeptidyl peptidase-1
EGFR: epithelial growth factor receptor
GM-CSF: granulocyte-monocyte colony-stimulating factor
ICS: inhaled corticosteroids
LABA: long-acting beta-2 agonist
LAMA: long-acting muscarinic antagonist
LTB4: leukotriene B4
MIF: migration inhibitory factor
MMP: matrix metalloproteinase
MPO: myeloperoxidase
NE: neutrophil elastase
NET: neutrophil extracellular trap
PAD4: peptidyl arginine deiminase 4
PAMP: pathogen-associated molecular pattern
PDE: phosphodiesterase
PR3: proteinase 3
RAGE: receptor for advanced glycation endproduct
RCT: Randomized Controlled Trial
T2: type-2
TLR: toll-like receptor
TNFα: tumor necrosis factor-α
TSLP: thymic stromal lymphopoietin
Th17: T helper 17
eDNA: extracellular DNA
sRAGE: signals via receptor for advanced glycation endproduct
